# Migrants’ invisible paths to mental health: the emergency department as gateway for psychiatric care

**DOI:** 10.1192/j.eurpsy.2026.10988

**Published:** 2026-07-31

**Authors:** A. Platini, G. Volta, N. F. De Luca, A. E. M. Bobbio, T. Lorenzetto, F. Gavelli, M. Bellan, C. Gramaglia, P. Zeppegno, E. Gambaro

**Affiliations:** 1 Università del Piemonte Orientale, Novara, Italy; 2 Translational Medicine, Università del Piemonte Orientale, NOVARA, Italy

## Abstract

**Introduction:**

Migration is a phenomenon which is increasing worldwide and is known to be a risk factor in the development of mental health issues, including psychotic disorders, especially among asylum seekers and refugees. High rates of anxiety disorders, depression and Post-Traumatic Stress Disorder are well documented, too. Due to lack of awareness of the host country’s healthcare system, migrants often turn to the emergency department (ED) which frequently becomes the entry point for mental healthcare due to stigma, delay in diagnosis and treatment.

**Objectives:**

The main goal of this study is to analyze socio-demographic and clinical features of migrants who sought care in the ED and were provided with a psychiatric consultation, in order to identify possible correlations with the outcome of such consultations and to compare them with those involving the native population.

**Methods:**

Data will be presented from the leading center (Maggiore della Carità University Hospital) of a larger project (IDEALI) which involves several Italian centers. Between January 2024 and March 2025, 91 migrants received a psychiatric consultation in the ED. For each patient socio-demographic and clinical data, as well as features related to ED access and subsequent discharge were collected and compared to those of the 483 Italian patients who received a psychiatric consultation in the same ED and in the same period of time.

**Results:**

Migrant patients accessed the ED mostly on their own or upon referral from their general practitioner/emergency medical service. The most frequent symptoms at presentation were anxiety and psychomotor agitation in both groups. Over the reference period, migrants accounted for only 16% of total ED visits, the majority of which resulted in hospitalization. The most frequent outcome for the psychiatric consultation was discharge or referral to a community mental health center. The majority of the sample had a history of psychiatric disorders, with psychotic and personality disorders being the most frequent diagnoses. Most migrants had previously undergone psychiatric treatments (usually benzodiazepines) and they received medication more frequently in the ED compared to non-migrants.

**Conclusions:**

This study confirms previous literature findings: migrants often rely on ED as the primary entry point to healthcare, including psychiatric services. Cultural and linguistic barriers complicate the access to community services and lead to a higher risk of urgent admissions, coercitive treatments and negative clinical outcomes. The high incidence of psychotic disorders observed in our sample highlights the urgent need to develop culturally competent and multidisciplinary care. Cultural-linguistic mediation, preventive strategies and strengthened continuity of care are essential to ensure equity and improve mental health outcomes in the migrant population.

**Disclosure of Interest:**

None Declared

